# Individualized Virtual Reality for Increasing Self-Compassion: Evaluation Study

**DOI:** 10.2196/47617

**Published:** 2023-10-02

**Authors:** Ilona Halim, Lehan Stemmet, Sylvia Hach, Richard Porter, Hai-Ning Liang, Atiyeh Vaezipour, Julie D Henry, Nilufar Baghaei

**Affiliations:** 1 School of Mathematical and Computational Sciences Massey University Auckland New Zealand; 2 Auckland Institute of Studies Auckland New Zealand; 3 Department of Psychological Medicine University of Otago Christchurch New Zealand; 4 Department of Computing Xi'an Jiaotong-Liverpool University Suzhou China; 5 Recover Injury Research Centre The University of Queensland St Lucia Australia; 6 School of Psychology The University of Queensland St Lucia Australia; 7 School of Electrical Engineering and Computer Science The University of Queensland St Lucia Australia

**Keywords:** individualized virtual reality, mental health, self-compassion, depression, depressive symptoms, mobile phone

## Abstract

**Background:**

Depression and anxiety are common and debilitating mental disorders with severe negative repercussions at both individual and societal levels. Although virtual reality (VR) has emerged as a safe and effective tool for the treatment of anxiety disorders, studies of the therapeutic application of VR to treat depression are more limited.

**Objective:**

The purpose of this study was to test whether a novel type of individualized VR (iVR) can be used to improve self-compassion and decrease depressive symptoms and to evaluate the usability and acceptability of this approach, as rated by participants. The iVR system was designed and developed based on the feedback obtained from a previous study, with improved appearance and feel of the avatar and enhanced graphical quality.

**Methods:**

A total of 36 young adult participants were recruited from a university community social media site. Participants were aware that the study was investigating a treatment for depression but were not recruited based on depression diagnosis. Participants were asked to complete 2 iVR sessions, spaced 2 weeks apart. At baseline and upon completion of each iVR session, participants were asked to complete validated measures of self-compassion and depression. Upon completion of both iVR sessions, additional measures were administered to assess participants’ perceptions about the perceived usability and system acceptability of the iVR approach.

**Results:**

Self-compassion was assessed at the beginning of session 1 (preintervention baseline) and at the end of session 1 (postintervention assessment). Owing to COVID-19 constraints, 36% (13/36) of the participants were unable to complete the follow-up iVR session. Self-compassion was assessed again for the remaining 64% (23/36) of the participants at the end of session 2 (postintervention assessment). Within-group analyses revealed that self-compassion was significantly increased at the end of both session 1 (*P*=.01) and session 2 (*P*=.03) relative to baseline. There was also a nonsignificant trend for depressive symptoms to be low at the end of session 2 relative to baseline. Both quantitative and qualitative participant data supported the iVR approach as being acceptable and usable.

**Conclusions:**

Although these data must be treated as preliminary owing to the small sample size and potential selection bias, the data provide encouraging initial evidence that iVR might be a useful tool to enhance self-compassion and reduce depressive symptoms, highlighting the need for randomized controlled trials in the future.

## Introduction

### Background

Depression is a common mental disorder and a leading cause of disability that has been identified by the World Health Organization as a major contributor to the overall global burden of disease [1]. Psychological and pharmacological treatments, such as cognitive behavioral therapy, interpersonal psychotherapy, and antidepressant medication, are commonly used to treat depression [1]. However, despite the availability of treatment, it has been estimated that between 76% and 85% of people in low- and middle-income nations do not obtain adequate care owing to lack of resources and trained health care professionals [1,2]. Although steps have been taken to try and overcome these obstacles, more effective and accessible care is still urgently required [1].

Studies suggest that there is a link between self-compassion or self-criticism and depressive symptoms, with the former considered as a protective factor and the latter as a risk factor for depression [3]. Such links are unsurprising, given that self-compassion involves treating oneself with kindness and understanding [4], whereas self-criticism is defined by negative self-evaluation and judgment [5]. Low self-compassion and high self-criticism are associated with more severe depressive symptoms and increased risk of chronic or recurring depression [6].

Importantly, studies suggest that self-compassion can be increased with training [7]. Compassion-focused therapy (CFT) is therefore a possible method to reduce symptoms of depression. With CFT, the aim of the treatment is to increase a person’s self-compassion levels through strategic exposure to situations or scenarios that encourage demonstration of compassion toward themselves or others and allow the client to focus on self-compassion when they experience negative thought processes [8]. However, although this method has been shown to be effective, it can be challenging and resource intensive to implement; thus, there is a need for innovative interventions that are more accessible and sustainable [9].

### Virtual Reality in Mental Health

Virtual Reality (VR) is an emerging technology that can simulate realistic and immersive experiences within a virtual world. VR head-mounted displays (HMDs) are becoming more affordable and accessible, and there is a growing interest in their potential use in psychotherapeutic interventions [10,11]. VR exposure therapy (VRET) is one of the most common VR applications in mental health [12-14]. This exposure therapy strategy takes advantage of the high degree of presence provided by VR environments. Recent studies indicate that VR can be effective in the treatment of a variety of mental disorders, pain management, and addiction, where gains from simulated virtual exposure therapy transfer to real-life situations [15].

VR has been used to provide exposure therapy in the treatment of phobia, where people are presented with a simulation of their source of fear in a safe and controlled situation [16]. Positive results have been reported in the treatment of acrophobia, trypanophobia, claustrophobia, and vehophobia and for more complex anxiety disorders including panic disorder, social anxiety disorder, and arachnophobia [17].

In particular, intensive studies have focused on the use of VR for the treatment of acrophobia (fear of heights). Krijn et al [18] investigated whether cognitive coping self-statements would have additional benefits over VRET in people with acrophobia. Participants (N=26) were randomly assigned to receive either 2 sessions of VRET and coping self-statements followed by 2 sessions of VRET or 2 sessions of VRET followed by 2 sessions of VRET and coping self-statements. Findings indicated that VRET reduced height-related anxiety and avoidance behavior, regardless of whether coping self-statements were added. In a study conducted by Meyerbroker et al [12], VRET was used to treat acrophobia and fear of flying. Findings showed that VRET with a placebo is an effective treatment for acrophobia. More recently, Donker et al [19] conducted a study on the use of a smartphone VR app for VRET. In total, 193 participants with acrophobia were randomly assigned to a VR cognitive behavioral treatment group using cardboard VR goggles (n=96, 49.7%) or to a waiting list control group (n=97, 50.3%). Results showed reduction in acrophobia symptoms after therapy and at 3-month follow-up.

In the treatment of people with posttraumatic stress disorder (PTSD), VR has also been used as a tool for exposure therapy by presenting simulated scenarios that trigger the trauma in combination with relaxation training [20]. Although prolonged exposure involving emotional processing of traumatic situations and habituation of anxiety is an evidence-based treatment for PTSD, some people are either unwilling to undergo this treatment or are unable to visualize the traumatic stimuli because of anxiety avoidance [21]. For people living with PTSD, exposure to a controlled fictitious virtual environment might therefore be more tolerable than exposure by imagination [21]. Several randomized controlled trials (RCTs) have compared VRET with prolonged imaginal exposure therapy in active-duty US military personnel and veterans. The VRET sessions included imaginal exposure to traumatic war memories and computer-generated environments, tailored to participants’ description of their trauma, and revealed that VRET was just as effective as extended exposure [22]. Although a large RCT comparing prolonged imaginal exposure with VRET in active-duty personnel found that prolonged exposure was more effective than VRET at 3-month and 6-month follow-up [23], Norr et al [24] found that relative to a waitlist control, both prolonged exposure and VRET led to decreased incidence of suicidal thoughts.

VR has also been used to assess mental health disorders, albeit less frequently. For instance, it has been used to simulate circumstances in which addiction-related behaviors might be triggered, so that the therapist can watch the client’s response and use that knowledge to plan the patient’s therapy [25]. Extensive literature shows that alcohol-related and drug-related cues (such as cigarettes, ashtrays, and smokers at a bar) contribute to the persistence of substance use disorders [26], and this extends to the virtual world. Moreover, peer pressure from virtual avatars is highly effective in inducing cravings [12,27]. Collectively, the findings from these studies show that immersing individuals in virtual worlds related to their specific addiction might be a valuable tool in both their assessment and treatment.

### VR in the Treatment of Depression

In a scoping study conducted by Baghaei et al [14], it was argued that VR could be beneficial in helping the treatment of anxiety or depression in a variety of contexts, and its potential as a clinical tool was highlighted. However, despite this emerging literature about the use of VR for mental health conditions, relatively few studies so far have focused on depression, one of the most common conditions [28]. Of the limited number of studies that have tested the potential value of VR in treating depression, the first study used the standard nonindividualized approach. Li et al [29] investigated the impact of a VR-based restorative environment on the emotional and cognitive recovery of patients with mild to moderate depression and anxiety. The study enrolled 195 individuals with mild to moderate depression and anxiety. The study used multiple VR restorative contexts, including an urban and a park setting. Participants were divided into groups at random and each engaged with a different scene. According to the findings of the study, a VR-based restorative environment can improve the emotional well-being and cognitive recovery of people with mild to moderate anxiety and depression. Falconer et al [28] examined the effect of avatar embodiment on self-compassion in people diagnosed with clinical depression. In this study, 15 participants engaged in an 8-minute VR simulation in which they experienced delivering and receiving compassion in virtual bodies. This session was repeated 3 times, and at the conclusion of the trial, 9 participants reported improvement in their depressive symptoms. Our group later followed up this study, by investigating whether *individualization* of VR might increase the potential benefits of compassion for depressive symptoms [30]. An early version of an individualized VR (iVR) system was built and implemented and was evaluated [31]. In this study, participants were able to individualize their VR experience by selecting their preferred avatar, therapeutic environment, and treatment scenario.

### Individualization of VR

The potential benefits of *personalizing* virtual experiences for mental health therapy, such as customizing the virtual environment to an individual’s preferences and interests, has been the focus of limited studies. Most VR therapies have been predefined and predesigned, resulting in a uniform approach for all participants based on an immersion or presence-specific environment [32]. Although this approach has the advantage of being standardized, it does not account for important individual differences in characteristics, which may influence the treatment’s efficacy [33]. iVR can also improve user experience and facilitate therapists’ access to otherwise difficult-to-obtain clinical information [14,31].

Avatars are common across digital applications. By using avatars as self-representation, we engage in social activities, play, and conduct businesses. There has been an increasing interest in the potential effects of avatar customization on both user experience and performance indicators [34]. Customization of avatars has been found to positively influence outcomes in a variety of fields. In players who personalized their game characters, increased identification with their avatars was found to increase autonomy, invested effort, enjoyment, and immersion [35]. Waltemate et al [36] examined the effect of the degree of personalization and individualization of users’ avatars and the level of immersion on typical psychophysical variables in embodied virtual environments. Personalized avatars in VR were found to enhance body ownership, sense of presence, and dominance in comparison with generic counterparts of comparable realism and quality of graphics. Such findings demonstrate the potential value of individualized avatars in VR applications that rely on body ownership and presence. As the architecture of iVR mainly relies on avatar embodiment [31,32], avatar customization should theoretically enhance the application. As therapists may also view the individual’s choice of avatar in each session, the type of avatar characteristics selected also provides further unique insights into their client’s mental state.

However, although the results from our previous study [31] provided important proof of concept in showing that individualization of the VR experience may have value in therapeutic contexts, this study did not examine the effects of iVR on self-compassion levels and depression symptoms specifically. Therefore, the central aim of this study was to report the design and implementation of a novel VR system that builds on but meaningfully improves the systems used in our early pilot study [31,32] and then to use this refined system to provide the first evaluation of the implemented iVR’s impact on self‑compassion and depressive symptoms.

### iVR Design and Implementation

#### iVR Description

Our prototype iVR system mirrors the prototype used by Falconer et al [28] and the iVR system prototype used in our initial pilot study [30]. The VR application consists of 2 phases. First, participants are instructed to show or give compassion to a crying child in a virtual environment. This is then followed by the second stage, in which participants are given the opportunity to assume the role of the crying child and receive compassion. The broad literature on compassion predicts that both giving and receiving compassion should increase self‑compassion.

In this study, individualization features were navigated via graphical user interface (GUI) panels added to the virtual environment throughout all stages. However, because qualitative feedback from participants indicated that participants wanted clear instructions about how to use the application, we then added a main menu or tutorial stage at the beginning of our iVR system. Therefore, the application includes three specific stages:

1. The main menu stage, which provides clear instructions about the application and enables participants to customize their experience
2. The delivering compassion stage, which allows participants to practice delivering compassion
3. The receiving compassion stage, which presents the opportunity to experience receiving compassion

#### Stage 1: Main Menu or Tutorial

In the main menu, participants are presented with a brief explanation of how the VR application works and are guided through the process of selecting their choice of avatar, environment, and scenario. To maintain consistency, all menu options in the main menu follow a template. The title of the menu is displayed at the top of the menu panel. All options are populated under the title banner.

**Figure 1:**
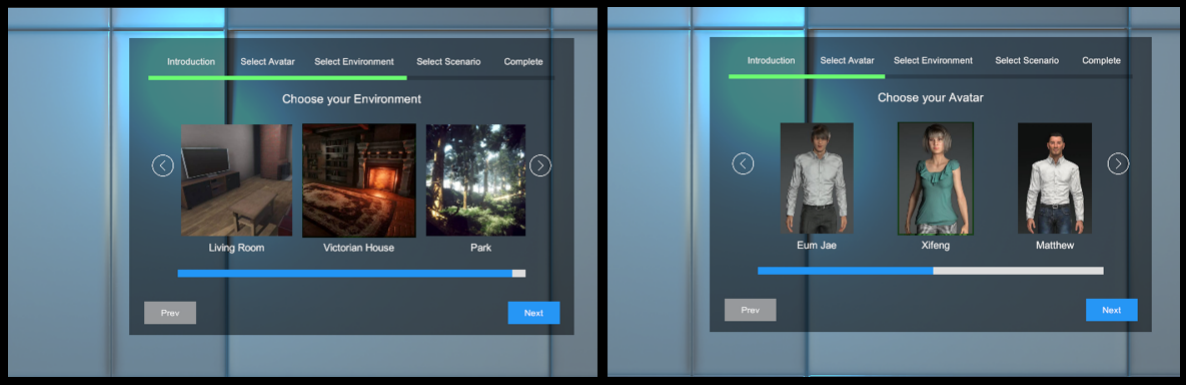
Main menu stage in the individualized virtual reality prototype.

The contents of the menu options vary between individualization categories. For instance, the avatar selection section might contain various virtual body selections, whereas the environment selection section might contain indoor and outdoor terrain selections. Figure 1 is a screenshot of the main menu panel as viewed by the participant. Interactions with the main menu occur via hand-held controllers. When participants have completed customizing their VR experience, they can then proceed to stage 2, where they can practice delivering compassion.

#### Stage 2: Delivering Compassion

##### Overview

In stage 2 of the prototype, participants are given the opportunity to deliver compassion to a distressed in-game character. Participants embody a virtual avatar when delivering compassion in a scene generated based on their previous choices. Unlike the predetermined virtual body in the prototype used by Falconer et al [28], the appearance of the body is determined based on the avatar chosen from the main menu stage. The therapy environment and the in-game character’s appearance and behavior can also be individualized. Participants were instructed to give compassion to a distressed youth closely following the 3-phase approach of *validation*, *redirection of attention*, and *memory activation*, which is the standard approach recommended for use in CFT when dealing with emotional situations [28].

##### Validation

The aim of this phase is to acknowledge that the other person is upset, that you do not judge them for this, and that it is perfectly acceptable for them to react in this way. The following is a sample dialogue: “It’s not nice when things happen to us that we don’t like. It has really upset you, hasn’t it?”

##### Redirection of Attention

The aim of this phase is to direct the other person’s attention toward something that is more positive, soothing, and comforting. The following is a sample dialogue: “Sometimes when we are sad it’s helpful to think of someone who loves us or is kind to us.”

##### Memory Activation

The aim of this phase is to suggest that the person could try to recall a memory of a person who loves or is kind to them. This memory is supposed to instill more positive feelings of warmth, comfort, and safety. The following is a sample dialogue: “Can you think of someone who loves you or is kind to you? What might they say to you now that would make you feel better?”

**Figure 2:**
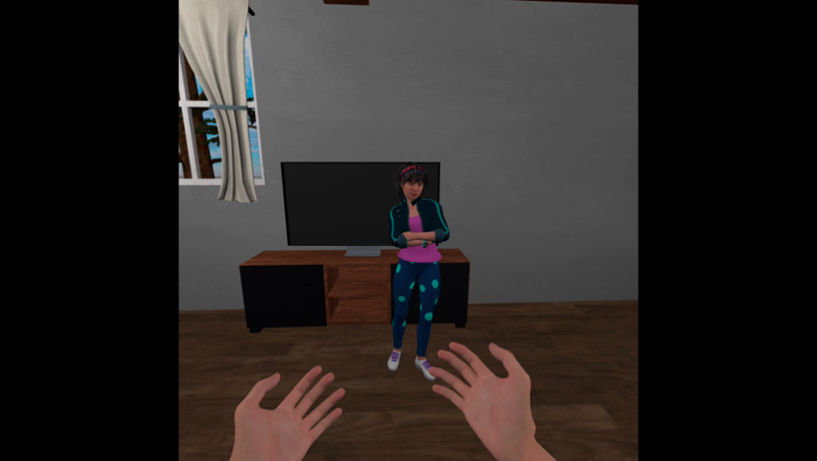
A screenshot of a participant delivering compassion in the individualized virtual reality prototype.

Hints and clear instructions were incorporated into the virtual environment to enhance user experience. When the stage is loaded, participants’ speech is automatically recorded. After delivering compassion, they can go to the next stage by clicking the “Next Stage” button. Figure 2 depicts a stage-1 scene in which a participant has decided to interact with an upset young female character by embodying an Asian female avatar in an indoor setting.

#### Stage 3: Receiving Compassion

**Figure 3:**
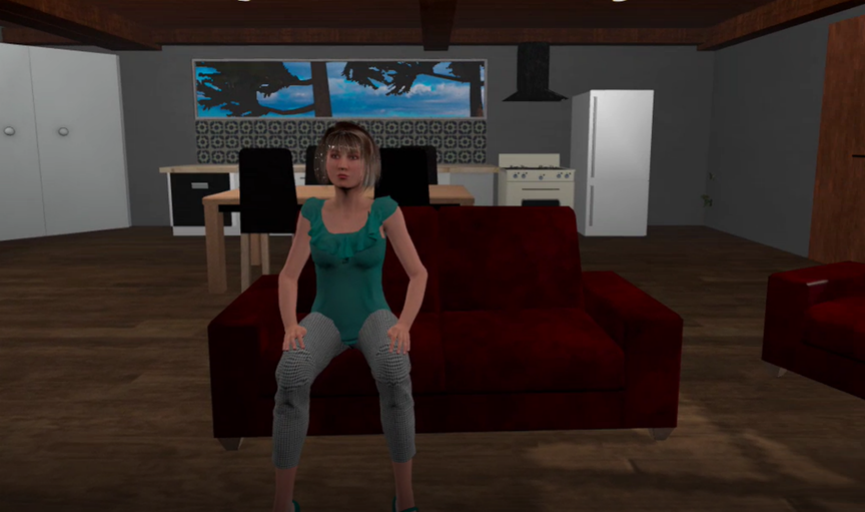
A screenshot of participant receiving compassion in the individualized virtual reality prototype.

Stage 3 gives users the opportunity to experience receiving compassion from their chosen virtual self through the eyes of the game character as illustrated in Figure 3. During this stage, participants hear a virtual avatar reenacting the participant’s speech that was recorded from the first stage to deliver compassion, but this time, they are on the receiving end of the speech. When the avatar has completed acting the scene, the scenario ends, and they are given the option of returning to the main menu or quitting the application.

### Individualized Components

#### Overview

The iVR application gives participants an opportunity to personalize their avatar, environment, and scenario. When used in a therapeutic environment, observing the client’s decisions could potentially assist therapists in providing additional clinical insights, particularly for clients who are not willing or able to provide accurate information about their mental health.

Although the customization of avatars in our early pilot study [31] was well received, feedback from participants revealed a preference to interact with avatars that were more graphically realistic. Therefore, improving the appearance and feel of the avatar was one of the primary objectives in building the iVR prototype.

Reallusion software (Reallusion Inc) [37] was used to produce preexisting multilayered humanoid components for this project. The degree of realism and graphic quality of these assets are relatively good. However, these files use single meshes for the skin layer, making it difficult to programmatically alter the avatar’s personal attributes, such as clothing and skin tone, based on participant’s choice. Owing to this, the prototype’s avatar customization was restricted to a selection of premade virtual bodies. As this prototype was intended for use in a New Zealand–based study, avatar options were made available for the region’s 3 largest ethnic groups: Asian, European or Pakeha, and Māori [38]. The available choices of virtual bodies are as follows:

1. Asian female
2. Asian male
3. European female
4. European male
5. Māori female
6. Māori male

#### Virtual Environments

In the early prototype [31], users could choose from a variety of therapeutic environments. However, these environments did not depict the landscape in a visually accurate manner. Participants showed a tendency to favor locations with the most realistic appearance because this enhanced their sense of immersion [31]. Our current iVR prototype design focuses on enhancing the graphical quality of the selected environments to make them more realistic and immersive.

It is important to strike a balance between performance and the complexity of digital resources when developing the virtual environment. High-fidelity resources can produce a more realistic-looking environment but can be detrimental to the performance of the application because it creates a great burden on the software and hardware of the VR HMD to load the digital models. This was one of the difficulties encountered during the development of iVR environments.

**Figure 4:**
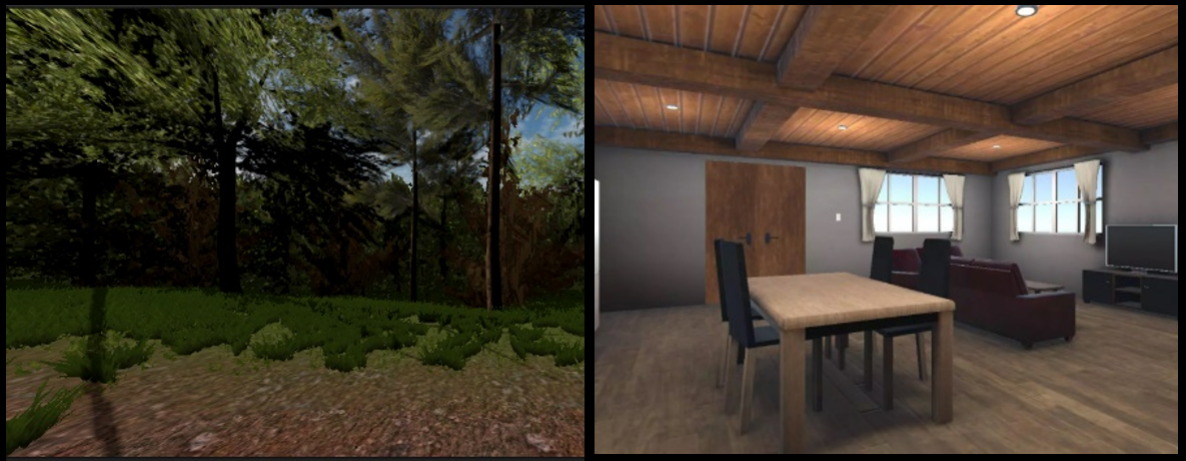
Improved (A) outdoor virtual environment and (B) indoor virtual environment used in the individualized virtual reality prototype.

To avoid performance issues, the iVR system was developed using photorealistic 3D digital models that were not so complex as to hinder the software’s performance. In addition, we used occlusion culling to increase productivity by avoiding the rendering of items that are obscured from view. Figure 4 depicts the iVR prototype’s enhanced assets in outdoor and indoor environments. We included both indoor and outdoor environments for the participants to choose from, including a living room, a study room in a Victorian house, and a park.

#### Interaction Scenario

In addition to being able to choose their own in-game character, participants could select from two potential scenarios:

1. Delivering compassion to a crying person
2. Delivering compassion to an angry or upset person

Creating a convincingly realistic humanoid model can be difficult because humans are graphically complex objects, and the human eye is adept at identifying subtle humanoid object characteristics [39]. Overall, three distinct types of strategies can be used to animate human behaviors: (1) keyframing, (2) motion capture, and (3) simulation [40], each with specific advantages and limitations [41]. The keyframing technique requires the key positions of objects in each timeline to be manually specified. Using this technique, animators may control the motion of an object’s minute elements. It also allows for a more seamless transition between stances. The disadvantage is that it can be time consuming. However, although the motion capture approach is typically less time consuming than the keyframe method, it does not provide much control over the motion and does not necessarily have seamless transitions between scenarios. The third method is simulation, in which lifelike movements are generated. However, this may be prohibitively expensive and time consuming, making it impractical at this time. After assessing the advantages and disadvantages of each technique, keyframing was chosen to provide great control and smooth scenario transitions.

### Design Considerations for VR Application

Immersive VR is fundamentally different from conventional digital products designed for flat 2D screens in that consumers receive a fully immersive 3D experience equivalent to the real world. Consequently, several aspects must be considered when designing components for a VR application.

#### Vision

When designing virtual components, the comfort of the user was the primary concern. According to the recommendations by Meta [42], all menus and displays must be within a comfortable sight distance to avoid eye strain. For most end users, 1 m is the best seeing distance for GUIs and menus [42]. Following this advice, the main menu and GUI panels were rendered approximately 1 m away from the user, as measured in a real-world setting.

Another key factor considered was the end users’ line of sight. Szauer [43] recommends that key gameplay items be placed within the immediate field of view of the user, as objects placed outside the user’s field of view are commonly overlooked. In the iVR prototype, avatars and virtual bodies were therefore positioned exactly in front of the camera, within its typical range of view.

**Figure 5:**
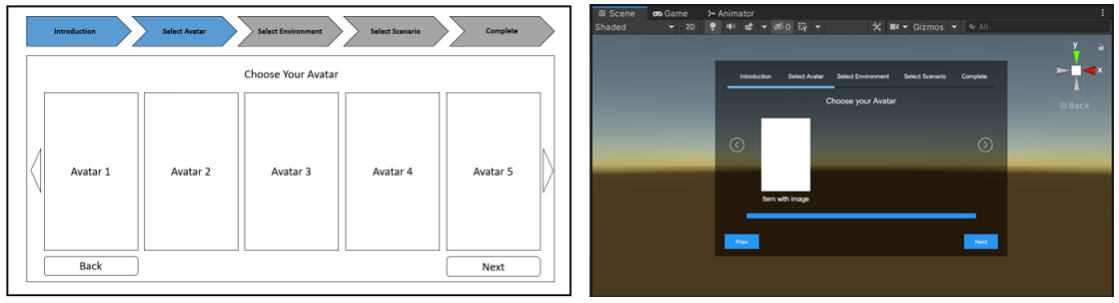
(A) Low-fidelity and (B) high-fidelity prototype of the avatar selection menu panel.

GUIs and menus must be drawn in front of the user’s eyes and positioned relative to the camera, so that they are always within the user’s field of view. All menu and user interface information must be displayed without requiring the user to move their head [43]. As we have various customizable settings, it was difficult to display all of them on a single panel. To address this issue, the menu options were divided into sections and subsequently shown using a paginated panel. The next or back button allows users to browse through the menu. Figure 5 illustrates a low-fidelity and high-fidelity prototype of the avatar selection menu panel.

For user input, this project used the Meta (old Oculus) Quest 2 controller input with laser pointers for GUI and menu interactions to provide participants with a more engaging and participatory experience.

#### Locomotion

In contrast to 2D displays, users can physically move around in a VR world. In VR development, the user’s motions within the virtual environment are typically referred to as locomotive. Comfortable locomotion has a direct and meaningful effect on the user experience, making it essential for the success of any VR application [44].

Physical and artificial locomotion are the 2 primary types of locomotion in the creation of VR [45]. Physical locomotion is when motions in the virtual world are synchronized with the user’s movements in the real world. Artificial locomotion is when motions in the virtual world are controlled by external factors, such as button presses, as opposed to physical movement. Although this can be advantageous for navigating the virtual environment when physical space is limited, certain artificial actions, such as smooth turning, can be uncomfortable [45]. Therefore, caution should be exercised when developing an artificial locomotive in the virtual world. Teleportation may be used to handle artificial movement in the virtual environment [44], but it was not incorporated in the iVR prototype because participants might not be familiar with the VR HMD, and this functionality was susceptible to user mistakes. Therefore, the iVR for mental health application was designed to allow users to navigate the virtual world by walking in the real world.

### Development Tools

The Unity [46] game engine was used in the development of the iVR prototype because it offers an intuitive user interface with a short learning curve. In addition, Unity provides flexible VR plugins that are compatible with Meta Quest 2.

**Figure 6:**
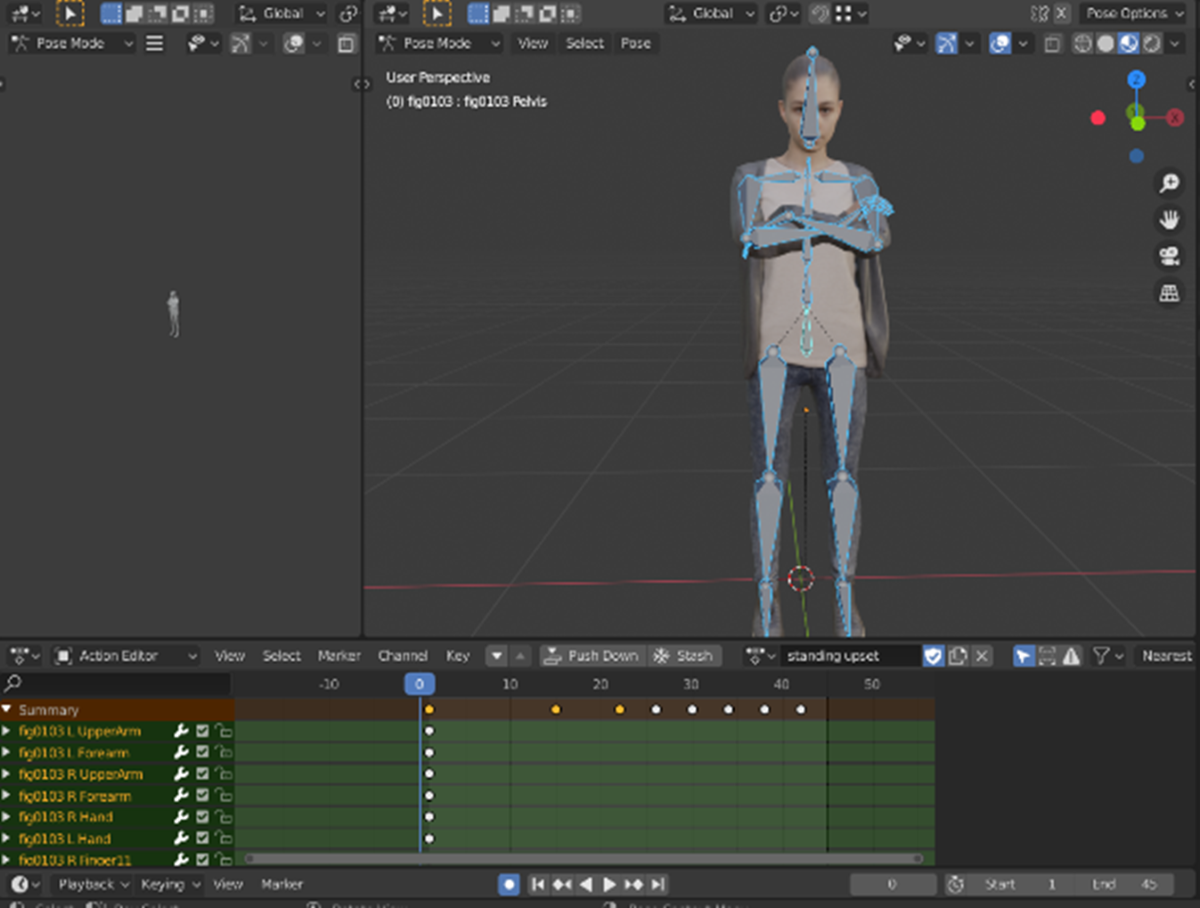
Keyframing animation process in Blender.

Virtual avatars in the iVR prototype were animated using Mixamo [47] and Blender [48]. First, Mixamo was used to produce generic movement patterns for the models. Upon completion of the animation process in Mixamo, the file was converted into a FilmBoX file, so that it may be used in the Unity project (as depicted in Figure 6). Blender was then used to make any necessary animation adjustments, and the file was then imported into Unity. The animation transitions and position orders were then defined in Unity using animation controllers.

The iVR prototype records the audio input of the Meta Quest 2 headset during stage 1 and uses the recording as the audio input for stage 2. Participants were able to hear and observe their avatar reenacting stage 1 from a different perspective. To control the facial expressions of the avatar, Oculus VR Lipsync (Meta) was used. Oculus VR Lipsync [49] is a Unity add-on plugin that may be used to synchronize the lip and facial movement of a digital object with audio input.

## Methods

The goal of the study was to examine whether iVR increases self-compassion and decreases symptoms of depression.

### Participants

A total of 36 participants agreed to participate (n=17, 47% self-identified as men and n=19, 53% self-identified as women), who were on average aged 29.3 (SD 11.64) years. Participants were recruited via poster advertising, social media advertising, and word of mouth. Participants were informed that it was a study about depression, but there was no requirement for participants to have a depression diagnosis and that they did not need to disclose whether they were clinically depressed. Only participants aged >18 years were allowed to participate in the study because it dealt significantly with sensitive topics related to mental health. Participants were also required to reside in Auckland, New Zealand, because the experiment needed to be conducted in person at a specific location. Potential participants who expressed interest through email were sent a copy of the information sheet, and they provided written informed consent before completing the experimental sessions. All participants were reimbursed with a gift card worth NZD $20 (US $11.8) upon completion of the study.

### Measures

#### Self-Compassion Scale

The Self-Compassion Scale (SCS) [50] was used to assess self-compassion. SCS is a tool created by Kristin Neff [51] to measure self-compassion. It is frequently used in academic settings [50]. SCS is a 26-item questionnaire that has previously been shown to be a reliable and valid indicator of self‑compassion [52]. High scores on SCS are indicative of great self-compassion. SCS data were collected from participants 3 times throughout the study: at the beginning (baseline preintervention assessment), at the end of the first session (after interaction with iVR), and at the end of the follow-up session (after interaction with iVR). To determine whether the iVR session had any impact on participants’ levels of self-compassion, the postintervention SCS scores were compared with the baseline score.

#### Patient Health Questionnaire

The 9-item Patient Health Questionnaire (PHQ-9) [53,54] and 8-item Patient Health Questionnaire (PHQ-8, which is an 8-item subset of PHQ-9) [53], have been widely used in both research settings and therapeutic trials [54]. PHQ-9 is a short self-report questionnaire that combines The Diagnostic and Statistical Manual of Mental Disorders depression diagnostic criteria [55] with additional prevalent major depressive symptoms. High scores on PHQ-9 or PHQ-8 are indicative of high depressive symptoms. PHQ-8 omits the question about suicidal and self-harm ideas that is occasionally used to gauge the risk of suicide from PHQ-9 [54]. For the purpose of major depressive disorder screening, PHQ-8 was found to be just as helpful as PHQ-9 because it is a sensitive indicator of depression symptomatology [56]. As this study did not specifically assess the risk of suicide, depression symptoms were measured using the PHQ-8 scale. PHQ-8 scores were collected at the beginning of the trial (preintervention baseline), at the end of the first session (after interaction with iVR), and at the end of the follow-up session (after interaction with iVR). The PHQ-8 scores after the intervention were then compared with the baseline score to see if the iVR session had any effect on participants’ depression symptoms.

#### System Usability Scale

The System Usability Scale (SUS) [57] is a 10-item, standardized questionnaire that was used to assess the perceived usability of VR systems by end users [57]. Previous studies have shown it is a valid or reliable indicator of VR system usability [58]. It has proven to be a robust tool, having been used to evaluate a wide range of interfaces, including websites, VR applications, GUI, and television user interfaces [57]. High scores on this measure are indicative of better usability.

#### User Experience Questionnaire

The User Experience Questionnaire (UEQ) [59] is a 26-item questionnaire that was used to evaluate the acceptability of the VR application. UEQ provides an index of a product’s pragmatic and hedonic quality aspects by considering 6 scales: attractiveness, perspicuity, efficiency, dependability, stimulation, and novelty [59].

#### Qualitative Feedback

Information about participants’ subjective preferences (what they liked and disliked about the iVR experience) and any potential recommendations they might have for improvement was also obtained upon the conclusion of the follow-up session. The following questions were asked: (1) What were the top three things that they liked about iVR? (2) What didn’t they like about iVR? and (3) How do you think the next version can be improved? What other features would you like to see? Participants’ responses were transcribed for qualitative analysis, using the thematic analysis method [60].

### Procedures

#### Overview

We conducted 2 one-on-one sessions for each participant, completed a minimum of 2 weeks apart. The first session took approximately 40 to 45 minutes, whereas session 2 took approximately 20 to 30 minutes to complete. After providing informed consent, in session 1, participants were first asked to complete the questionnaires about depression and self-compassion (PHQ-8 and SCS). After completing the surveys, individuals were given the opportunity to become comfortable with the experimental setup and the VR technology. If they were unfamiliar with VR HMDs, participants were asked to watch a video example of the experimental process and received basic instructions about how to use the Meta Quest 2 VR HMD. In addition, they received an instruction sheet that explained the idea of CFT and the pertinent steps (validation, attentional redirection, and memory activation). They were then asked to interact with the iVR application for approximately 6 to 10 minutes. The participants interacted with the iVR application in 3 stages as previously outlined in the iVR Design and Implementation section.

#### Stage 1: Main Menu or Tutorial (Approximately 2 min)

This stage involves the following:

1. Interaction with GUI panels to choose the virtual avatar, environment, and scenario.

#### Stage 2 Delivering Compassion (Approximately 2 min)

This stage involves the following:

1. Visuomotor synchrony or embodiment with the chosen avatar through movement
2. Interaction with crying, angry child, or youth
3. Instruction: “React to the child/youth by using the sentences you have learned just now.”

#### Stage 3 Receiving Compassion (Approximately 2 min)

This stage involves the following:

1. Visuomotor synchrony or embodiment with crying, angry child, or youth through movement
2. Real-time playback of recorded interaction part B from the perspective of crying, angry child, or youth
3. Instruction: “Stand/sit, look and listen.”

Participants were allowed to explore the system without restriction, with researchers present in the room to assist if necessary. After the interaction, they were asked to complete the same measures of depression and self-compassion again, in addition to measures of their experience of engaging with the VR system (SUS and UEQ). In session 2, participants commenced the session by interacting with the iVR application and then were asked to complete the measures of depression and self-compassion and provide qualitative feedback about the iVR application.

### Ethics Approval

The study was approved by Massey University’s Human Research Ethics Committee (NOR 21/83).

### Analyses

Although a total of 36 participants completed session 1, owing to COVID-19 constraints only 23 (64%) participants were able to complete session 2. A post hoc power analysis conducted using G*Power revealed that for the key comparisons of interest (whether there was any significant change in self‑compassion and depression upon completion of session 1 relative to baseline and upon completion of session 2 relative to baseline), our within-participants design with 23 participants still had sufficient power (>0.80) to detect a moderate to large effect difference (Cohen *d*=0.65) at Cronbach α=.05 (2 tailed), for each of these comparisons. Owing to the dependent nature of the samples, paired-samples 2-tailed *t* tests were used for these key contrasts.

## Results

### Mental Health Data Following the iVR Intervention

Descriptive statistics for both SCS and PHQ-8 are reported in Table 1.

**Table 1:** Descriptive statistics for the key measures of mental health (N=36).

Measure	Baseline		Session 1 (postintervention assessment)		Session 2 (postintervention assessment)	
	Score, mean (SD)	Participants, n (%)	Score, mean (SD)	Participants, n (%)	Score, mean (SD)	Participants, n (%)
SCS^a^	2.94 (0.68)	35 (97)	3.07 (0.73)	35 (97)	3.23 (0.80)	23 (64)
PHQ-8^b^	8.52 (6.32)	23 (64)	7.44 (6.15)	23 (64)	6.70 (4.97)	23 (64)

^a^SCS: Self-Compassion Scale.

^b^PHQ-8: 8-item Patient Health Questionnaire.

For both SCS and PHQ-8, skewness fell within an acceptable range to permit parametric data analysis [61]. A series of paired-sample 2-tailed *t* tests were therefore used to analyze the changes in SCS and PHQ-8 scores. Regarding SCS, these analyses revealed that, relative to baseline, there was a significant increase in participants’ self-compassion, both at the end of session 1 (t_34_=2.64; *P*=.01) and at the end of session 2 (t_22_=2.37; *P*=.03). Regarding PHQ-8, the same analyses revealed that, relative to baseline, self-rated depression was overall unchanged at the end of session 1 (t_35_=1.69; *P*=.10) but that there was a nonsignificant trend for depressive symptoms to be low at the end of session 2 (t_22_=1.88; *P*=.07).

### Associations Between Self-Compassion and Depressive Symptoms

The next step in the analyses was to calculate Pearson correlations [62] to test how participants’ self-compassion and depressive symptoms related to one another across the different stages of the study. These correlations are reported in Table 2. There were significant negative associations between SCS and PHQ-8 scores at all time points assessed, and the magnitude of these associations was consistently large [63].

**Table 2:** Correlation analysis of the 8-item Patient Health Questionnaire (PHQ-8) and Self-Compassion Scale (SCS) scores.

Variables			SCS (baseline)		SCS (session 1)		SCS (session 2)		PHQ-8 (baseline)		PHQ-8 (session 1)		PHQ-8 (session 2)
**SCS (baseline)**													
	*r*	1		0.949		0.747		–0.743		–0.699		–0.76	
	*P* value	—^a^		<.001		<.001		<.001		<.001		<.001	
**SCS (session 1)**													
	*r*	0.949		1		0.775		–0.741		–0.694		–0.727	
	*P* value	<.001		—		<.001		<.001		<.001		<.001	
**SCS (session 2)**													
	*r*	0.747		0.775		1		–0.581		–0.531		–0.735	
	*P* value	<.001		<.001		—		.004		.009		<.001	
**PHQ-8 (baseline)**													
	*r*	–0.743		–0.741		–0.581		1		0.955		0.685	
	*P* value	<.001		<.001		.004		—		<.001		<.001	
**PHQ-8 (session 1)**													
	*r*	–0.699		–0.694		–0.531		0.955		1		0.64	
	*P* value	<.001		<.001		.009		<.001		—		.001	
**PHQ-8 (session 2)**													
	*r*	–0.76		–0.727		–0.735		0.685		0.64		1	
	*P* value	<.001		<.001		<.001		<.001		.001		—	

^a^Not applicable.

### iVR System Usability and Acceptability

Data collected from 36 participants upon completion of session 1 revealed that the overall SUS score for the iVR application was 75.9 (SD 13.09). According to the adjective rating system defined by Bangor et al [57], this falls within the good to excellent range.

**Figure 7:**
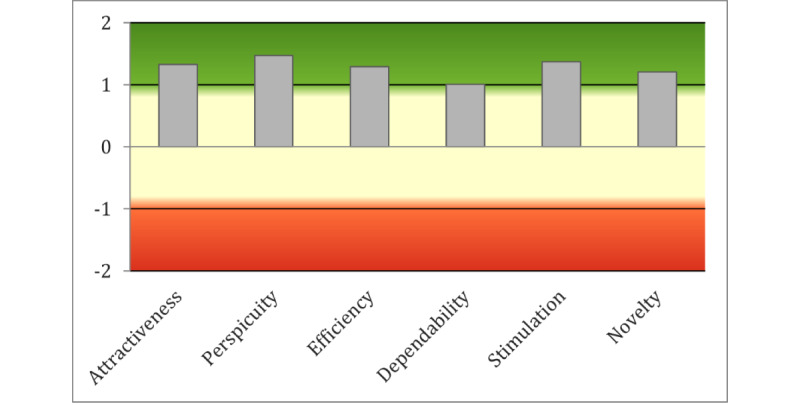
User Experience Questionnaire results.

UEQ data were also gathered from 36 participants at the end of session 1. Figure 7 presents the ratings for the 6 UEQ scales.

**Figure 8:**
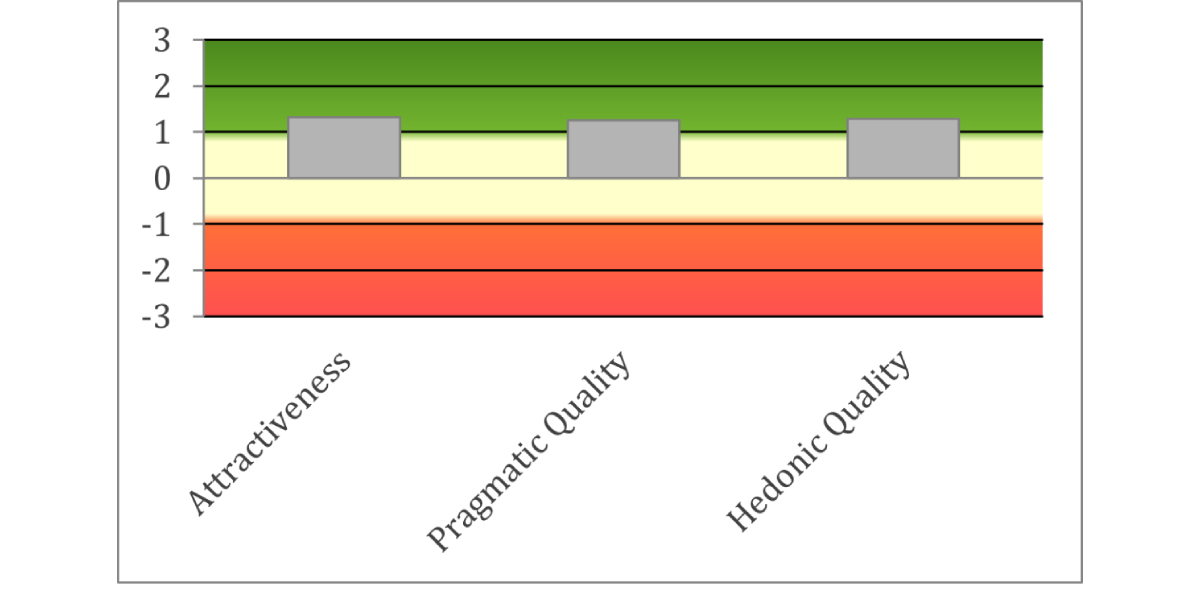
User Experience Questionnaire results grouped by attractiveness, pragmatic quality, and hedonic quality.

Figure 8 shows the mean of the UEQ scales grouped by attractiveness, pragmatic quality (perspicuity, efficiency, and dependability), and hedonic quality (stimulation and originality).

**Figure 9:**
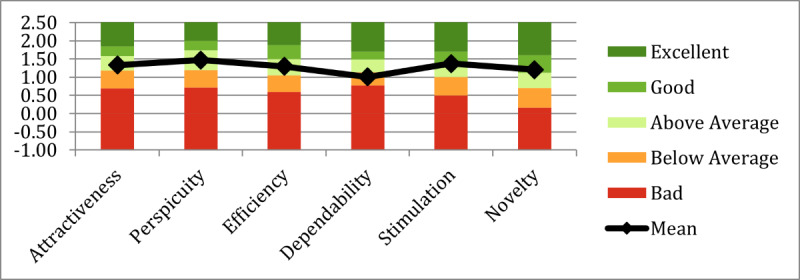
User Experience Questionnaire results in comparison with benchmark data.

The UEQ analysis tool [59] was used to compare the UEQ results with a benchmark data set containing data from 20,190 individuals from 452 studies of different products. Figure 9 presents the results of the comparison.

Relative to the benchmark data, the iVR prototype’s UEQ scores for attractiveness and efficiency lie within the “Above Average” range, whereas scores for perspicuity, novelty, and stimulation fall within the “Good” range. However, dependability falls within the “Below Average” area of UEQ measures.

Finally, qualitative feedback was gathered from 64% (23/36) of the participants at the end of session 2. Thematic analysis was used to interpret these responses [60]. This revealed that participants’ responses to the question, “What were the top three things that they liked about iVR?” could be categorized according to the following themes:

1. The top element that participants liked was that it is user-friendly. Approximately half of the participants (17/36, 47%) liked how the app was “easy to use.” Other quotes included the following: “easy instructions,” “easy navigation,” and “easy to use and has a positive vibe.”
2. Participants liked how they could customize their VR experience. Quotes from participants included the following: “choose personal avatar,” “I enjoyed customizing my situation,” “how you got to choose your person and environment,” and “I liked that you can pick different areas.”
3. Participants liked how the experience felt real and immersive. Quotes included the following: “The immersive aspect really added an element of connection,” “It feels like a person is there with you,” and “Illusion of real experience.”
4. Participants liked the use of VR technology. Quotes included the following: “I like virtual reality,” “VR is very immersive,” “suitable for VR because other systems are just a single screen,” and “I just enjoy VR.”
5. Some participants liked the virtual environment. Quotes included the following: “calming environment,” “The background was calming,” and “loved the scenery.”
6. Some participants liked the user interface design. Quotes included the following: “Clean UI” and “I liked that the menu was simple.”

For the question, “What didn’t they like about iVR?” answers from participants could be grouped into the following:

1. Participants disliked the limited interaction with the virtual avatars. Quotes included the following: “Maybe there can get more operation to do,” “Very dull, nothing really happened,” and “a bit more interaction and maybe some more prompts.”
2. Some participants found that the system was a little slow. Quotes included the following: “Laggy, sometimes unresponsive,” “has some lags,” “I found the VR menu was a bit slow,” and “A little laggy going from one phase to the next.”
3. Some participants felt that the scenarios were very simple. Quotes included the following: “The scenario could be more complex” and “I wish it had more scenarios.”

The responses to the question, “How do you think the next version can be improved? What other features would you like to see?” can be categorized as follows:

1. Participants suggested adding more robust scenarios to choose from. Quotes included the following: “You could create different scenarios and paths for users in the next version,” “Maybe a more interesting scenario, like an argument,” and “Perhaps expanding on the first stage to include more situations.”
2. Participants would also like to see more interactions with the virtual avatars and virtual environment. Quotes included the following: “more interaction with the environment,” “More interaction, such as we can walk together, have a seat,” “Better user interaction,” and “The AI reacting to the user.”
3. Some participants wanted more avatar options to be added. Quotes included the following: “more avatars and settings” and “More diverse ethnic avatars.”
4. Some participants wanted to see improved graphics and a more natural avatar. Quotes included the following: “I’d like if the character models moved a bit more natural.”
5. Some participants said that they wanted to see improved performance of iVR. Quotes included the following: “Improve load speed and fps.”

## Discussion

### Principal Findings

The findings of this study provide important novel insights into the potential value of iVR as a tool to increase participants’ self-compassion and reduce their depressive symptoms and about the usability and acceptance of the iVR system in general. Although these findings are preliminary, it is hoped that they will help to motivate future studies in this area.

#### Changes in Self-Compassion Level and Depressive Symptoms

The most noteworthy finding was the significant increase in participants’ self-compassion after interacting with the iVR prototype. There was a significant increase in participants’ self-compassion after as little as a single session of iVR. These findings suggest that iVR could potentially be a helpful tool to improve self-compassion. As noted previously, the iVR session was designed to explicitly incorporate elements of CFT by encouraging participants to engage in compassionate behavior and compassion-based skills. Therefore, this study provides further support to the broad literature showing that self-compassion can be increased by training [7] and extends it meaningfully by showing that these benefits also emerge when this training is provided in the virtual world.

Regarding depression, participants’ depressive symptoms were unchanged at the end of session 1 relative to baseline, but there was a nonsignificant trend for their symptoms to be reduced at the end of session 2. We consider these data to be encouraging, particularly considering the nonclinical nature of the cohort. This is because many participants reported relatively low levels of depressive symptoms at baseline. Thus, an important area of future studies is to use this novel iVR approach with participants who present with clinically significant levels of depressive symptomatology

Another important finding to emerge was that depressive symptoms and self-compassion were significantly correlated with one another at all stages of the study, and the magnitude of these associations was consistently large [63]. Although the design of this study clearly precludes inferences about causality, increased self-compassion should theoretically help decrease depression symptoms. Therefore, the finding of large, robust associations between measures of these 2 constructs broadly align with the broad literature that has established self-compassion as a protective factor against depression [5].

#### Usability and Acceptability of the iVR Prototype

In addition to showing that the iVR application was associated with significantly enhancing self-compassion, participants also generally found it to be highly usable. The mean SUS score for the iVR application was 75.9, which falls within the good to excellent range [57], indicating that participants mostly perceived that the system has good usability.

Comparing the UEQ ratings with the values in the benchmark data set was also valuable in highlighting the iVR prototype’s specific strengths and weaknesses. Novelty and stimulation were rated as “Good” on UEQ, outperforming 75% of the systems within the benchmark. This suggests that participants viewed the iVR prototype as innovative and captivating. However, the dependability scale of UEQ was graded as “Below Average,” indicating that participants may not experience a sense of control when interacting with the iVR system. Moreover, some participants believed the system to be slow. This suggests that there may be performance issues with the iVR prototype, and future iterations of iVR should optimize system performance and speed to improve the user experience.

### Limitations and Future Directions

Despite the encouraging results of this study, there are several limitations that create opportunities for future studies. First, COVID-19–related travel restrictions and people testing positive for COVID-19 meant that many participants were unable to attend their follow-up appointments, and this made an RCT unfeasible.

Although our reduced sample size in session 2 still had adequate power to detect moderate to large within-group differences, obviously, such a small cohort is unlikely to be representative of the broad population. The absence of a control condition also means that we cannot rule out the possibility that the positive effects identified were related to nonspecific factors other than the iVR sessions. Such factors include expectation effects, natural resolution of depressive symptoms over time, and positive interactions with the research team. COVID-19 lockdowns and restrictions could also be a major influence on depressive symptoms, as shown in studies conducted during the pandemic [64,65]. Therefore, to cross-validate our results, the next important step is a large-scale RCT that includes participants presenting with clinically significant levels of depressive symptoms. It would also be very valuable to include a long-term follow-up to establish how long any observed gains persist.

Owing to the small sample size, this study was also underpowered to assess the potential influence of variables such as gender, culture, and ethnicity. Overt features, such as gender and ethnic and cultural disparities [33], can have a substantial impact on VR interventions and contribute to variance in results, even when the same VR simulation treatment is used. Therefore, consideration of such variables in iVR designs represents another important area for future studies.

Another limitation of our design was that we did not investigate VR variables, such as presence and embodiment. Future studies of iVR may benefit from considering these variables, as presence in the virtual environment and sense of embodiment toward the avatar may moderate the effects of VR interventions. For instance, according to Grassini et al [66], presence may influence training outcomes. It is noteworthy that, in this study, participants’ qualitative data indicated that they enjoyed how immersive the VR environment was and how it made them feel as if they were there in person. It remains to be established whether presence was an important determinant of the efficacy of the intervention.

Comparison of the results of qualitative participant feedback about their recommendations for future iterations of iVR with those of previous studies [31] also revealed a few recurring themes that might be explored for future iterations of the iVR. For instance, more robust avatar interaction and additional avatar options should be added to potentially enhance the user experience. In a future study with a large sample size, it would also be interesting to examine the link between usability scores and depressive symptoms to establish if those with severe depression symptoms have a different user experience from those with few symptoms.

Finally, it is also worth noting that the system only supported limited individualization. It would be of considerable value to test the impact of more advanced individualization (such as changing skin tone, hair color, and style) on participants’ self-compassion and depressive symptoms. We were also unable to capture physiological cues to record participants’ stress level or emotional states, and this also remains as an important avenue for future studies to build on the current findings.

### Conclusions

In this paper, we have outlined the design and implementation of an iVR application to enhance self-compassion and improve symptoms of depression. The pilot user study revealed that, relative to baseline, self-compassion increased after both a single and a second iVR session and that there was a nonsignificant trend for depressive symptoms to decrease after a second iVR session. Importantly, most users thought that the product’s usability was good or excellent.

Moving forward, we are evaluating potential improvements to the iVR application that would involve integrating more robust participant–virtual environment interaction, with emphasis on interactions with the avatars that are already present in the environment. We are considering including some interactive elements to boost user engagement. Adding artificial intelligence to the avatar is another option that is being considered. Our hope is that this study has the potential to pave the path for future, cost-effective, clinical applications and large-scale efficacy assessment of intelligent, iVR technology that might deliver mental health therapy in a safe, cost-effective, and engaging manner.

## Abbreviations

CFT: compassion-focused therapy
GUI: graphical user interface
HMD: head-mounted display
iVR: individualized virtual reality
PHQ-8: 8-item Patient Health Questionnaire
PHQ-9: 9-item Patient Health Questionnaire
PTSD: posttraumatic stress disorder
RCT: randomized controlled trial
SCS: Self-Compassion Scale
SUS: System Usability Scale
UEQ: User Experience Questionnaire
VR: virtual reality
VRET: virtual reality exposure therapy
